# Supplementary material for: Prediction of future customer needs using machine learning across multiple product categories
Source: PLoS One. 2024 Aug 26;19(8):e0307180. doi: 10.1371/journal.pone.0307180 (PMC11346667; doi:10.1371/journal.pone.0307180)
Supplement: S1 Appendix — (PDF) [file pone.0307180.s001.pdf]

## Appendix A Reddit Information Based Series

For the Reddit Information Based Series, we record 18 features, as shown in Table S1. These account for 51 of the univariate time series in the classification task i.e. 26 boolean based series (i.e. 13 features times 2 summary statistics); 2) 12 continuous based series (i.e. 3 features times 4 summary statistics); and 3) 13 string based series (i.e. 5 plus 8 type matches).

The string features we analyze are 1) *thumbnail* and 2) *whitelist_status*. Specifically we report the percent of times *thumbnail* equals a) "self", b) "default", c) "nsfw", d) "image" and e) "spoiler" and *whitelist_status* equals "all_ads", b) "no_ads", c) "some_ads", d) "promo_adult_nsfw", e) "house_only", f) "promo_all", g) "promo_adult" and h) "promo_specified". We search the following values across both the mentioned fields (e.g. self, default etc. for *thumbnail*) as they are an exhaustive list of the values we found in all the Reddit post data we collect.

**Table S1.** Reddit Features Used in Analysis

| **Name** | **Type** | **Num Series** | **Name** | **Type** | **Num Series** | **Name** | **Type** | **Num Series** |
|---|---|---|---|---|---|---|---|---|
| is_robot_indexable | bool | 2 | locked | bool | 2 | pinned | bool | 2 |
| is_original_content | bool | 2 | no_follow | bool | 2 | num_comments | cont | 4 |
| is_reddit_media_domain | bool | 2 | over_18 | bool | 2 | num_crossposts | cont | 4 |
| is_self | bool | 2 | send_replies | bool | 2 | score | cont | 4 |
| is_video | bool | 2 | spoiler | bool | 2 | thumbnail | str | 5 |
| is_crosspostable | bool | 2 | stickied | bool | 2 | whitelist_status | str | 8 |
