# Supplementary material for: Prediction of future customer needs using machine learning across multiple product categories
Source: PLoS One. 2024 Aug 26;19(8):e0307180. doi: 10.1371/journal.pone.0307180 (PMC11346667; doi:10.1371/journal.pone.0307180)
Supplement: S2 Appendix — (PDF) [file pone.0307180.s002.pdf]

Appendix B Frequency Based Series

For the Frequency Based Series, we record 4 keyphrase-level features, as shown in Table S2. As these are keyphrase-level statistics they result in 4 univariate time series, as detailed in Section 3.3.

As seen in the table, the difference between the *Document Frequency* and the *Relative Document Frequency* is that the *Document Frequency* reports the number of posts the keyphrase appeared in the Fixed Time Window (i.e. month). The *Relative Document Frequency* on the other hand, reports the total number of posts the keyphrase appeared in divided by the number of posts in the Fixed Time Window (i.e. month).

For the *Document Frequency Compared to a Background Corpus* field, we use the chi-square test [1] for the purposes of distinguishing if there is a significant difference between the keyphrase's expected frequency on Reddit compared to its observed frequency in a large-scale background reference corpus. As in [2–4], the test is computed for each keyphrase using a 2-by-2 contingency table in which a chi-square test statistic is returned. As this statistic only measures if there is a difference between the observed and expected frequency (i.e. high values for big differences and close to zero for small differences), we multiply the statistic by minus 1 if the expected frequency is greater than the observed frequency. We do this in order to distinguish that the observed frequency is greater than the expected frequency (or vice-versa) to the classification algorithm. As in [5], we use the python library *wordfreq* [6] (representative of a normal distribution of words) to act as our background corpus.

Table S2. Frequency Based Features Used in Analysis

**Name**	**Type**	**Num Series**	**Name**	**Type**	**Num Series**
Document Frequency	keyphrase-level	1	Document Frequency Compared to a Background Corpus	keyphrase-level	1
Relative Document Frequency	keyphrase-level	1	% Posts which are Submissions	keyphrase-level	1

References

1. Pearson K. X. On the criterion that a given system of deviations from the probable in the case of a correlated system of variables is such that it can be reasonably supposed to have arisen from random sampling. The London, Edinburgh, and Dublin Philosophical Magazine and Journal of Science. 1900;50(302):157–175.

2. Paquot M, Bestgen Y. Distinctive words in academic writing: A comparison of three statistical tests for keyword extraction. In: Corpora: Pragmatics and discourse. Brill Rodopi; 2009. p. 247–269.

3. Palomino MA, Wuytack T. Unsupervised extraction of keywords from news archives. In: Language and Technology Conference. Springer; 2009. p. 544–555.

4. Rayson P. Corpus analysis of key words. The encyclopedia of applied linguistics. 2012;.

5. Kilroy D, Healy G, Caton S. Using Machine Learning to Improve Lead Times in the Identification of Emerging Customer Needs. IEEE Access. 2022;10:37774–37795.

6. Speer R. rspeer/wordfreq: v3.0; 2022. Available from: https://doi.org/10.5281/zenodo.7199437.
