# Supplementary material for: Prediction of future customer needs using machine learning across multiple product categories
Source: PLoS One. 2024 Aug 26;19(8):e0307180. doi: 10.1371/journal.pone.0307180 (PMC11346667; doi:10.1371/journal.pone.0307180)
Supplement: S3 Appendix — (PDF) [file pone.0307180.s003.pdf]

Appendix C Product Information Based Series

For the Product Based Series, we record 6 continuous features, as shown in Table S3. These 6 continuous features result in 24 univariate time series when summarized, as described in Section 3.3. We initially run various pretrained models distinguishing different types of product information (e.g. buy intent, purchase intent etc.) over the posts to generate features. For each of the models we report the probability value of the post being associated with the output class, therefore making it a continuous value (e.g. 0.98) rather than a boolean (e.g. True).

We run 3 separate pretrained text classification models over the posts from the python library *Hugging Face* [1] in order to generate the features seen in Table S3: 1) *Sell/Buy Intent*, 2) *Purchase Intent* and 3) *Review Helpfulness*. For the *Sell/Buy Intent* features, the pretrained model tries to classify posts into either having "selling" or "buying" intent. An example of buying intent includes "I am looking for the purple ombre dress with floral bodice in a size 12 for my wedding in June this year" while a sample of selling intent includes "Boiler over 7 years old".[1] For *Purchase Intent*, we generate features from a *RoBERTa*-based model [2] which is fine-tuned on a dataset of 2000 purchase-intent and non-purchase-intent documents [3].[2] For the *Review Helpfulness* features, we use a model trained on a dataset of customer reviews from Amazon which contains an output label of an Amazon helpfulness score [4].[3]

We used a pretrained zero-shot model from *Hugging Face* so to generate the 3 remaining features: 1) *Discuss Product Features*, 2) *Discuss Product Ideas* and 3) *Discuss Customer Needs*.[4] Zero-shot classification is a learning paradigm that aims to train a model at predicting instances belonging to an unseen class [5]. We provide the model with unseen labels so to generate more features for our task. Specifically, we provide the model with the following class names: 1) product feature (i.e. *Discuss Product Features*), 2) product idea (i.e. *Discuss Product Ideas*) and 3) customer need (i.e. *Discuss Customer Needs*).

Table S3. Product Based Features Used in Analysis

Name	Type	Num Series	Name	Type	Num Series
Sell/Buy Intent	cont	4	Discuss Product Features	cont	4
Purchase Intent	cont	4	Discuss Product Ideas	cont	4
Review Helpfulness	cont	4	Discuss Customer Needs	cont	4

References

1. Wolf T, Debut L, Sanh V, Chaumond J, Delangue C, Moi A, et al. Transformers: State-of-the-art natural language processing. In: Proceedings of the 2020 conference on empirical methods in natural language processing: system demonstrations; 2020. p. 38–45.

[1] https://huggingface.co/obsei-ai/sell-buy-intent-classifier-bert-mini - last accessed 10/07/2024
[2] https://huggingface.co/j-hartmann/purchase-intention-english-roberta-large - last accessed 10/07/2024
[3] https://huggingface.co/banjtheman/distilbert-base-uncased-helpful-amazon - last accessed 10/07/2024
[4] https://huggingface.co/cross-encoder/nli-distilroberta-base - last accessed 10/07/2024

2. Liu Y, Ott M, Goyal N, Du J, Joshi M, Chen D, et al. Roberta: A robustly optimized bert pretraining approach. arXiv preprint arXiv:190711692. 2019;.

3. Hartmann J, Heitmann M, Schamp C, Netzer O. The power of brand selfies. Journal of Marketing Research. 2021;58(6):1159–1177.

4. Gamzu I, Gonen H, Kutiel G, Levy R, Agichtein E. Identifying helpful sentences in product reviews. arXiv preprint arXiv:210409792. 2021;.

5. Wang W, Zheng VW, Yu H, Miao C. A survey of zero-shot learning: Settings, methods, and applications. ACM Transactions on Intelligent Systems and Technology (TIST). 2019;10(2):1–37.
