# Supplementary material for: Prediction of future customer needs using machine learning across multiple product categories
Source: PLoS One. 2024 Aug 26;19(8):e0307180. doi: 10.1371/journal.pone.0307180 (PMC11346667; doi:10.1371/journal.pone.0307180)
Supplement: S4 Appendix — (PDF) [file pone.0307180.s004.pdf]

Appendix D Sentiment Based Series

For the Sentiment Based Series, the 28 features we record are all shown in Table S4. These 28 continuous features result in 112 univariate time series when summarized, as described in Section 3.3. For the single pretrained sentiment model we run over the Reddit posts, we report the probability value of the post being associated with the output class, therefore making it a continuous value (e.g. 0.98) rather than a boolean (e.g. True).

The pretrained sentiment model we use in our study is from *Hugging Face* [1]. It is a fine-tuned version of a *XtremeDistilTransformers* model [2] which is run over the *GoEmotions* dataset [3].[5] The *GoEmotions* dataset itself contains 28 output classes each representing a feeling/sensation e.g. *Anger*, *Caring*, *Disappointment*, *Excitement* etc. These output classes make up the features in Table S4.

Table S4. Sentiment Based Features Used in Analysis

**Name**	**Type**	**Num Series**	**Name**	**Type**	**Num Series**	**Name**	**Type**	**Num Series**
Admir -ation	cont	4	Disapp -roval	cont	4	Nervous -ness	cont	4
Amuse -ment	cont	4	Disgust	cont	4	Neutral	cont	4
Anger	cont	4	Embarr -assment	cont	4	Optim -ism	cont	4
Annoy -ance	cont	4	Excite -ment	cont	4	Pride	cont	4
Approval	cont	4	Fear	cont	4	Realiz -ation	cont	4
Caring	cont	4	Gratitude	cont	4	Relief	cont	4
Confusion	cont	4	Grief	cont	4	Remorse	cont	4
Curiosity	cont	4	Joy	cont	4	Sadness	cont	4
Desire	cont	4	Love	cont	4	Surprise	cont	4
Disapp -ointment	cont	4						

References

1. Wolf T, Debut L, Sanh V, Chaumond J, Delangue C, Moi A, et al. Transformers: State-of-the-art natural language processing. In: Proceedings of the 2020 conference on empirical methods in natural language processing: system demonstrations; 2020. p. 38–45.

2. Mukherjee S, Awadallah AH, Gao J. XtremeDistilTransformers: Task Transfer for Task-agnostic Distillation; 2021.

3. Demszky D, Movshovitz-Attias D, Ko J, Cowen A, Nemade G, Ravi S. GoEmotions: A Dataset of Fine-Grained Emotions. In: 58th Annual Meeting of the Association for Computational Linguistics (ACL); 2020.

[5] `https://huggingface.co/bergum/xtremedistil-l6-h384-go-emotion` - last accessed 10/07/2024
