# Supplementary material for: Prediction of future customer needs using machine learning across multiple product categories
Source: PLoS One. 2024 Aug 26;19(8):e0307180. doi: 10.1371/journal.pone.0307180 (PMC11346667; doi:10.1371/journal.pone.0307180)
Supplement: S5 Appendix — (PDF) [file pone.0307180.s005.pdf]

Appendix E Question Detection Based Series

For the Question Detection Based Series, the 5 features we record are all shown in Table S5. These 5 continuous features result in 20 univariate time series when summarized, as detailed in Section 3.3. For the pretrained models we run over the Reddit posts, we report the probability value of the post being associated with the output class, therefore making it a continuous value (e.g. 0.98) rather than a boolean (e.g. True).

The first model we run tries to detect whether a post is asking a question or stating an answer i.e. question answer model. We use a pretrained model for this task from *Hugging Face* which is trained on a dataset of questions and statements from *Kaggle*.[6][7] This generates the two features in Table S5: a) *Question* and b) *Statement*.

The second model we run tries to detect whether a statement is true (*Entailment*), false (*Contradiction*), or undetermined (*Neutral*) in the way it is expressed i.e. Natural Language Inference (NLI). The pretrained model we use for this is also from *HuggingFace* [1].[8] This generates three features in Table S5: a) *Entailment*, b) *Contradiction* and c) *Neutral*.

Table S5. Question Based Detection Features Used in Analysis

**Name**	**Type**	**Num Series**	**Name**	**Type**	**Num Series**
Question	cont	4	Contradiction	cont	4
Statement	cont	4	Neutral	cont	4
Entailment	cont	4			

References

1. Bhargava P, Drozd A, Rogers A. Generalization in NLI: Ways (Not) To Go Beyond Simple Heuristics; 2021.

[6]https://huggingface.co/shahrukhx01/bert-mini-finetune-question-detection - last accessed 10/07/2024

[7]https://www.kaggle.com/stefanondisponibile/quora-question-keyword-pairs - last accessed 10/07/2024

[8]https://huggingface.co/prajjwal1/bert-mini-mnli - last accessed 10/07/2024
