# Supplementary material for: Prediction of future customer needs using machine learning across multiple product categories
Source: PLoS One. 2024 Aug 26;19(8):e0307180. doi: 10.1371/journal.pone.0307180 (PMC11346667; doi:10.1371/journal.pone.0307180)
Supplement: S6 Appendix — (PDF) [file pone.0307180.s006.pdf]

Appendix F Embedding Based Series

For the Embedding Based Series, we record 2 main types of features, as shown in Table S6. These 2 main types of features can be split up into 75 continuous features for *Document Embeddings* (i.e. 300 univariate time series) and 50 keyphrase-level features for *Phrase Embeddings* (i.e. 50 univariate time series) resulting in 350 univariate time series when summarized, as detailed in Section 3.3. In this section, we generate embeddings on the post (or document) and keyphrase level.

Table S6. Embedding Based Features Used in Analysis

Name	Type	Num Series
Document Embeddings	cont	300
Phrase Embeddings	keyphrase-level	50

For the post-level embeddings (i.e. *Document Embeddings*), we use the python libraries *spaCy* [1] and *SBERT* [2]. Specifically, we use the *en_core_web_lg* model from *spaCy* (as used in Section 3.2) and the *all-MiniLM-L6-v2* model from *SBERT*.[9] The *en_core_web_lg* and the *all-MiniLM-L6-v2* models produce 300 and 384 dimensional embeddings retrospectively. As each dimension produced by the model needs to be summarized as a continuous feature (as described in Section 3.3), this leads to major increases in the running time of the experiment if we were to summarize all of the features produced by the model.

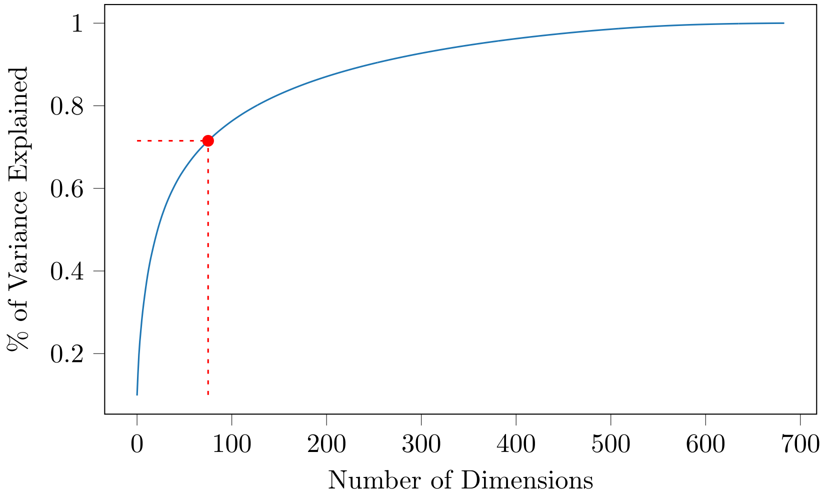

Fig S1. Percentage of Explained Variance Plot for PCA run over Document Embeddings - 75 components kept capturing 71.5% of the variance

In order to mitigate this time complexity issue, we run Principal Component Analysis (PCA) on the outputs of the embedding models and use the fitted model to transform new input data to a lower dimensional space. Specifically, we fit a PCA model on the document embeddings output of the *SBERT* and *spaCy* models concatenated. We do this as there are correlations between the two embedding outputs. We follow the suggested approach provided by *SBERT* when training the PCA model which recommends training on the transformed embedding output of 20,000 random

[9] `https://www.sbert.net/docs/pretrained_models.html` - last accessed 10/07/2024

documents from the *ALLNLI* dataset.[10] The *ALLNLI* dataset represents a highly general corpus - which is a combinations of the *SNLI* [3] and *MultiNLI* datasets [4].[11] Fig S1 shows a proportion of variance explained plot for the PCA model run over the concatenated document embeddings, which shows the % of variance explained on the y-axis and the number of dimensions on the x-axis. We select 75 dimensions to use in our analysis as it seems to provide a good proportion of the variance at a low number of components (as seen in Fig S1). To recap, when generating *Document Embedding* series, we first concatenate the document embeddings produced by the *SBERT* and *spaCy* models. We then transform the embeddings using the discussed trained PCA model and take the first 75 components. These 75 new features are then summarized to form 300 univariate time series (detailed in Section 3.3).

We also record embeddings on the phrase level by using pretrained word vectors from the python library *fasttext* [5]. Specifically, we use the *crawl-300d-2M-subword* model from *fasttext* which produces 300 dimensions.[12] The model is very useful has it can generate an embedding for any keyphrase as it uses subword information in its training. This can be helpful when dealing with the diverse range of phrases and misspellings on Reddit. As with the *Document Embeddings*, PCA is applied in order to retrieve a lower number of dimensions for time complexity purposes. Fig S2 shows a proportion of variance explained plot for the PCA model run over the phrase embeddings, which shows the % of variance explained on the y-axis and the number of dimensions on the x-axis. We selected 50 dimensions to use in our analysis as it seemed to provide a good proportion of the variance at a low number of components (as seen in Fig S2). To recap, when generating *Phrase Embedding* series, we first generate an embedding for the candidate keyphrase using the described *fasttext* model. We then transform the embeddings using the discussed PCA model and take the first 50 dimensions. Unlike the *Document Embeddings*, these embeddings are calculated on the keyphrase-level and are hence not summarized (detailed in Section 3.3).

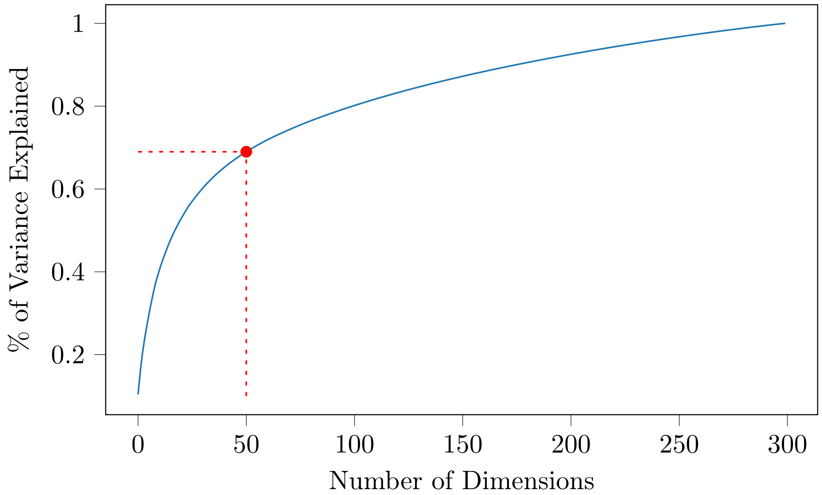

Fig S2. Percentage of Explained Variance Plot for PCA run over Phrase Embeddings - 50 components kept capturing 69% of the variance

[10] https://github.com/UKPLab/sentence-transformers/blob/master/examples/training/distillation/dimensionality_reduction.py - last accessed 10/07/2024

[11] https://www.sbert.net/examples/datasets/README.html#allnli-dataset - last accessed 10/07/2024

[12] https://fasttext.cc/docs/en/english-vectors.html - last accessed 10/07/2024

References

1. Honnibal M, Montani I, Van Landeghem S, Boyd A. spaCy: Industrial-strength natural language processing in python. Zenodo, Honolulu, HI, USA. 2020;.

2. Reimers N, Gurevych I. Sentence-BERT: Sentence Embeddings using Siamese BERT-Networks. In: Proceedings of the 2019 Conference on Empirical Methods in Natural Language Processing. Association for Computational Linguistics; 2019.Available from: https://arxiv.org/abs/1908.10084.

3. Bowman SR, Angeli G, Potts C, Manning CD. A large annotated corpus for learning natural language inference. arXiv preprint arXiv:150805326. 2015;.

4. Williams A, Nangia N, Bowman SR. A broad-coverage challenge corpus for sentence understanding through inference. arXiv preprint arXiv:170405426. 2017;.

5. Mikolov T, Grave E, Bojanowski P, Puhrsch C, Joulin A. Advances in Pre-Training Distributed Word Representations. In: Proceedings of the International Conference on Language Resources and Evaluation (LREC 2018); 2018.
