# Supplementary material for: Prediction of future customer needs using machine learning across multiple product categories
Source: PLoS One. 2024 Aug 26;19(8):e0307180. doi: 10.1371/journal.pone.0307180 (PMC11346667; doi:10.1371/journal.pone.0307180)
Supplement: S7 Appendix — (PDF) [file pone.0307180.s007.pdf]

Appendix G Subreddit Based Series

For the Subreddit Based Series, we record 100 search string features. As these are search string features they result in 100 univariate time series, as detailed in Section 3.3.

Specifically, we search for 100 subreddit strings from the most subscribed subreddits at the time of experimentation. These subreddits are found on a website containing an updated list of the most subscribed subreddits.[13] The names of these subreddits are shown in Table S7.

Table S7. Subreddit Names used as Search Strings in the analysis

Name	Name	Name	Name
announcements	DIY	wallstreetbets	dadjokes
funny	mildlyinteresting	wholesomememes	AnimalsBeingBros
AskReddit	sports	AdviceAnimals	tattoos
gaming	space	interestingasfuck	buildapc
aww	gadgets	Fitness	photography
Music	Documentaries	politics	AnimalsBeingJerks
pics	tifu	WTF	nba
worldnews	photoshopbattles	oddlysatisfying	BikiniBottomTwitter
science	GetMotivated	travel	Damnthatsinteresting
todayilearned	UpliftingNews	lifehacks	MadeMeSmile
movies	listentothis	Minecraft	FoodPorn
videos	television	relationship_advice	instant_regret
news	memes	facepalm	gardening
Showerthoughts	dataisbeautiful	BlackPeopleTwitter	reactiongifs
EarthPorn	history	NatureIsFuckingLit	AnimalsBeingDerps
food	philosophy	Whatcouldgowrong	woahdude
IAmA	InternetIsBeautiful	leagueoflegends	WatchPeopleDieInside
askscience	Futurology	bestof	Overwatch
Jokes	WritingPrompts	pcmasterrace	mildlyinfuriating
gifs	OldSchoolCool	me_irl	PewdiepieSubmissions
nottheonion	nosleep	dankmemes	programming
LifeProTips	personalfinance	nextfuckinglevel	PublicFreakout
books	creepy	Tinder	pokemon
explainlikeimfive	TwoXChromosomes	PS4	ContagiousLaughter
Art	technology	Unexpected	EatCheapAndHealthy

[13] `https://redditlist.com/` - last accessed 10/07/2024
