# Supplementary material for: Prediction of future customer needs using machine learning across multiple product categories
Source: PLoS One. 2024 Aug 26;19(8):e0307180. doi: 10.1371/journal.pone.0307180 (PMC11346667; doi:10.1371/journal.pone.0307180)
Supplement: S8 Appendix — (PDF) [file pone.0307180.s008.pdf]

Appendix H Kansei Engineering Based Series

For the Kansei Engineering Based Series, we record 32 boolean features, as shown in Table S8. As these are boolean statistics with no Not a Number (NaN) values they result in 32 univariate time series, as detailed in Section 3.3.

As detailed in Section 3.4, in order to integrate knowledge from Kansei Engineering into our model we identify 16 Kansei attributes. Each Kansei attribute makes up 2 features in our model, as each consists of 2 sets of bipolar words where users select their feelings towards a product e.g. common and unique. We follow the entire process from [1], to identify these sets of words i.e. 16 Kansei attributes which make up 32 sets of words. The initial words in each set are obtained from the literature. These words are then expanded by finding synonyms in each set. They are further expanded by finding antonyms in each of the bipolar groups which are then added to the corresponding group for the Kansei attribute e.g. add antonyms for "common" to the "unique-personalized-rare" group. In order to find these synonyms and antonyms we use Wordnet [2] using the python library *NLTK* [3]. We also follow the defined rules in [1] for resolving conflicts when a new word appears in two opposing Kansei groups. We then use these expanded lists of Kansei groups in order to classify a post as being associated with it, by virtue of the post containing a word in one of the Kansei groups. As stated, this results in 32 boolean features added to each post which creates 32 univariate time series as there are no NaN values present when calculating this feature. To note, every single row in Table S8 shows a Kansei attribute with the left column usually donating a positive group and the right a negative group (e.g. *Elegant* vs *Artless* belong to two different groups but the same attribute).

Table S8. Kansei Based Features Used in Analysis

**Name**	**Type**	**Num Series**	**Name**	**Type**	**Num Series**
Elegant	bool	1	Artless	bool	1
Simple	bool	1	Complex	bool	1
Comfortable	bool	1	Restrained	bool	1
Classic	bool	1	Hi-tech	bool	1
Soft	bool	1	Hard	bool	1
Loose	bool	1	Coarse	bool	1
Quality	bool	1	Unreliable	bool	1
Personalized	bool	1	Common	bool	1
Stylish	bool	1	Traditional	bool	1
Luxurious	bool	1	Low-cost	bool	1
Portable	bool	1	Bulky	bool	1
Pleasant	bool	1	Unpleasant	bool	1
Fresh	bool	1	Boring	bool	1
Practical	bool	1	Useless	bool	1
Bright	bool	1	Dim	bool	1
Professional	bool	1	Amateur	bool	1

References

1. Wang WM, Li Z, Tian Z, Wang J, Cheng MN. Extracting and summarizing affective features and responses from online product descriptions and reviews: A Kansei text mining approach. Engineering Applications of Artificial Intelligence. 2018;73:149–162.

2. Fellbaum C. WordNet. In: Theory and applications of ontology: computer applications. Springer; 2010. p. 231–243.

3. Loper E, Bird S. Nltk: The natural language toolkit. arXiv preprint cs/0205028. 2002;.
