# Supplementary material for: Prediction of future customer needs using machine learning across multiple product categories
Source: PLoS One. 2024 Aug 26;19(8):e0307180. doi: 10.1371/journal.pone.0307180 (PMC11346667; doi:10.1371/journal.pone.0307180)
Supplement: S9 Appendix — (PDF) [file pone.0307180.s009.pdf]

Appendix I Linguistic Based Series

For the Linguistic Based Series, we record 38 features, as shown in Table S9. These result in 456 univariate time series, as detailed in Section 3.3. As discussed in Section 3.4 the families of linguistic features we record are: 1) tagging information 2) document information and 3) phrase-level information. For most of the features recorded in this section we use the *en_ core_ web_ lg* model from spaCy [1], as used in Section 3.2.

For tagging, we record information related to Part-of-speech (POS) tags, dependency labels and named entities. When recording POS information, we consider tags provided by *spaCy*, which are in the Universal Dependencies format [2] and the Penn Treebank format [3]. The Universal Dependencies format records general POS tags (e.g. verbs or adjectives) while the Penn Treebank format records POS information in a more in depth manner (e.g. gerund verbs and comparative adjectives).[14] As these POS tags are strings we perform string matching in order to generate features which are later turned into univariate time series, as described in Section 3.3. The chosen strings we match for in the "*POS tags (Uni Dep)*" field (i.e. Universal Dependencies format) and the "*POS tags (Penn Treebank)*" field (i.e. Penn Treebank format) are 1) verbs, 2) adjectives, 3) nouns, 4) proper nouns and 5) adverbs. We do this as these are the only POS tags we accept when generating candidate keyphrases, as described in Section 3.2. Specifically, we search for all combinations of the Universal Dependencies tag list format (which contains a total of 5 tags) and the Penn Treebank tag list format (which contains a total of 17 tags) within an ngram range of 1-2 grams. An example of a one-gram POS string could be a single verb while a two-gram POS string may be a verb-adjective pair. Combinations of these tags generate 30 new univariate series for the "*POS tags (Uni Dep)*" field (i.e. 25 two-gram strings plus 5 one-gram strings) and 306 new series for the "*POS tags (Penn Treebank)*" field (i.e. 289 two-gram strings plus 17 one-gram strings). We don't search across an ngram range above 2 grams as it would result in too many new features which would lead to a considerable increase in computational complexity when classifying time series e.g. a three-gram range for the *POS tags (Penn Treebank)* field would result in 2744 more univariate series. Due to this, we change the way we search for strings by instead including a string as a match if it is contained as a subset of a searched string e.g. a noun-noun-verb string would be matched by a searched noun-noun string. When searching for dependency labels (i.e. *Dep tags* field), we search across all of the 45 labels provided by spaCy which are trained on OntoNotes 5.0 [4].[15] We only search for direct dependency labels and not co-occurring ones (as with POS tags) as doing it for two-grams would add a total of 2025 more univariate time series. For the same reasons as with POS tags, it is considered a match if it contains a subset. *SpaCy* also performs Named Entity Recognition (NER) and provides tagging for its 18 named entities i.e. the *NER tags* field. These entities are searched for and added as univariate series into the model. SpaCy also includes the Inside Outside Beginning (IOB) tags of these recorded entities (i.e. the *IOB tags* field) - these tags are also searched for and added as series.

For the document level linguistic information, we record general statistical information about the document e.g. how many tokens are in the post. Information about a lot of these series can be found in spaCy's *span* documentation.[16]

For the phrase-level linguistic information, we record general statistical information about each phrase e.g. number of vowels. Information about a lot of these series can be

[14] These POS labels can be accessed under token.pos_ (Universal Dependencies) and token.tag_ (Penn Treebank) in spaCy - `https://spacy.io/usage/linguistic-features#pos-tagging` - last accessed 10/07/2024

[15] `https://spacy.io/models/en#en_core_web_lg` - last accessed 10/07/2024

[16] `https://spacy.io/api/span` - last accessed 10/07/2024

found in spaCy's *token* documentation.[17] Some additional simple phrase-level information is manually created i.e. *num_vowels*, *contains_@*, *contains_#*, *num_tokens*, *contains_original* (i.e. contains the corresponding Target Keyphrase in Table 2 - "lip balm" for a dataset of lip balm products) and *contains_original_subset* (i.e. contains part of the corresponding Target Keyphrase in Table 2 - "lip" for a dataset of lip balm products). The features made manually are created using simple logic e.g. the binary feature *contains_@* is created by string searching for an @ symbol.

Table S9. Linguistic Features Used in Analysis

Name	Type	Num Series	Name	Type	Num Series	Name	Type	Num Series
POS tags (Uni Dep)	string	30	contains_non_lower	bool	1	contains_space	key-phrase	1
POS tags (Penn Treebank)	string	306	contains_oov	bool	1	contains_stop	key-phrase	1
Dep tags	string	45	length_text	cont	4	contains_email	key-phrase	1
IOB tags	string	3	token_index	cont	4	contains_num	key-phrase	1
NER tags	string	18	char_index	cont	4	contains_url	key-phrase	1
end	cont	4	contains_non_alpha	key-phrase	1	contains_@	key-phrase	1
end_char	cont	4	contains_non_ascii	key-phrase	1	contains_#	key-phrase	1
start	cont	4	contains_currency	key-phrase	1	length_lemma	key-phrase	1
start_char	cont	4	contains_digit	key-phrase	1	num_vowels	key-phrase	1
contains_upper	bool	1	contains_left_punct	key-phrase	1	num_tokens	key-phrase	1
contains_title	bool	1	contains_punct	key-phrase	1	contains_original_subset	key-phrase	1
contains_sent_start	bool	1	contains_quote	key-phrase	1	contains_original	key-phrase	1
contains_sent_end	bool	1	contains_right_punct	key-phrase	1			

[17] `https://spacy.io/api/token` - last accessed 10/07/2024

References

1. Honnibal M, Montani I, Van Landeghem S, Boyd A. spaCy: Industrial-strength natural language processing in python. Zenodo, Honolulu, HI, USA. 2020;.

2. Nivre J, De Marneffe MC, Ginter F, Goldberg Y, Hajic J, Manning CD, et al. Universal dependencies v1: A multilingual treebank collection. In: Proceedings of the Tenth International Conference on Language Resources and Evaluation (LREC'16); 2016. p. 1659–1666.

3. Santorini B. Part-of-speech tagging guidelines for the penn treebank project (3rd revision). Technical Reports (CIS). 1990; p. 570.

4. Weischedel R, Palmer M, Marcus M, Hovy E, Pradhan S, Ramshaw L, et al. Ontonotes release 5.0 ldc2013t19. Linguistic Data Consortium, Philadelphia, PA. 2013;23.
