# Supplementary material for: Prediction of future customer needs using machine learning across multiple product categories
Source: PLoS One. 2024 Aug 26;19(8):e0307180. doi: 10.1371/journal.pone.0307180 (PMC11346667; doi:10.1371/journal.pone.0307180)
Supplement: S10 Appendix — (PDF) [file pone.0307180.s010.pdf]

Appendix J User Based Series

For the User Based Series, we record 8 features, as shown in Table S10. These features result in 114 univariate time series, as detailed in Section 3.3.

The types of features we record for users is guided from the literature which can be divided into two broad categories: 1) personal user information; 2) user interaction information. For personal information, the types of features include publicly listed details on the user's profile e.g. gender, age, location, number of friends/followers/connections, etc. [1, 2]. For interaction information, users are linked to form a network graph using some type of relation (e.g. follower/followee). Statistics are then calculated on the graph to measure the way in which users interact e.g. number of edges, strength distribution, shortest path, etc. [2]. In our analysis, it's challenging to create features for authors due to the Pushshift Application Programming Interface (API) not yet providing access to user-level information.[18] In comparison to Pushshift (i.e. historical API), the general Reddit API also doesn't provide a lot of personal user information at the time of experimentation e.g. age, gender, location etc.[19] It also has a limit fives time less than Pushshift [3]. This makes extracting information from it infeasible from a time complexity perspective as there are users coming from more than 4 million posts in our study which will need to have data collected for them. Therefore when creating personal and interaction information for users in our study we are restricted in the features we generate.

For the personal user information, we generate different types of features other than publicly listed details on the user's profile (as discussed). Specifically, we mine the author's username as it's some of the only personal information available for users through the Pushshift API. However, in addition to not being able to extract a lot of user information from Reddit, it's also difficult to infer attributes from authors usernames as done in previous studies e.g. predict gender from usernames [4]. This is because authors on Reddit use fake names (e.g. Stuck_In_the_Matrix) instead of real ones [5]. Due to the mentioned factors, the way in which we generate user-based features differs slightly from the norm. Although it's not their real name, we do a basic analysis of the username field provided with each post when generating features about the personal information of Reddit authors. Specifically, we search for defined strings in usernames to extract high-level information about them. Some of these defined strings may include "bot" or "mod" (as in [6]) which are commonly put in the names of internet robots and moderators respectively. It'd be useful to distinguish these types of authors as they don't discuss customer needs but rather post spam [7, 8] or point out lapses in other authors "reditiquette" [9]. Specifically, we generate the mentioned searched strings automatically instead of defining ones we think are of interest e.g. bot or mod. We do this as we want a data-driven way to find the most common substrings in authors usernames instead of guessing them ourselves. To find these substrings, we collect author usernames from 20,000 random Reddit posts using Pushshift.[20] Across these usernames, we search for the 100 most common substrings 3-8 character n-grams in length. To search for these substrings using the following parameters we use the Bag-of-Words model in *sklearn*.[21] For the n-gram range (i.e. 3-8), we choose a minimum of 3 n-grams as we want the substrings to at least capture some high-level character information (e.g. not 2-grams like "as" or "an") and a maximum of 8 n-grams for the

[18] As of 12/04/2023 Pushshift has not developed an endpoint to "analyze a Reddit user's activity" - `https://github.com/pushshift/api#list-of-endpoints` - last accessed 10/07/2024

[19] `https://praw.readthedocs.io/en/stable/code_overview/models/redditor.html` - last accessed 10/07/2024

[20] These collected posts are independent of the main data collection process (described in Section 3.1)

[21] `https://scikit-learn.org/stable/modules/generated/sklearn.feature_extraction.text.CountVectorizer.html` - last accessed 10/07/2024

upper range as most substrings don't exceed 8 grams.[22] We choose the total number of substrings to be 100 as we want to keep the number of substring searches low for computational purposes. From the list of character substrings we generate some pertain to interesting topics. Two include "man" and "her" which are helpful in inferring the gender of Reddit authors. In fact, this use of character-level n-gram information from usernames has been used to predict gender on social media before [10–12]. Another example from the list includes the substring "red", which may infer other information about the authors. A complete list of Reddit username substrings can be found in the GitHub repository which accompanies this study (recorded in Section 1). As described in Section 3.3, we generate 100 univariate time series from these substrings by matching strings in the author's username associated with each Reddit post.

For the user interaction information, we link authors using a network graph for a different type of relation other than some measure of friendship (e.g. follower/followee/friend), as this information is not available through the Pushshift API. Specifically, we instead form a graph by linking authors by the subreddits they post in i.e. subreddit graph. We then calculate graph-based statistics which make up the interaction features. For all the statistics we calculate on the network graph in this study, we use the python library *networkx*.[23] To calculate statistics for this information we create two graphs: 1) *All Posts Graph* and 2) *Sub Posts Graph*. The *Sub Posts Graph* is a subgraph of the *All Posts Graph*. Graph-based statistics are calculated on the *Sub Posts Graph* which make up the the user-interaction features in this study. The two graphs along with the features are calculated each Fixed Time Window as with all the other features in this study (discussed in Section 3.3). When creating the *All Posts Graph*, we first take all the posts in the Fixed Time Window and link the authors from the subreddits they post in. We link authors this way due to restrictions on accessing user-level information through the Reddit API's. From these interactions, we form an undirected graph where the nodes are authors and the edges are chosen based on if the users post in the same subreddit during the Fixed Time Window of analysis. We use an undirected graph as if the authors post in the same graph they are deemed to be connected, which is unlike a situation in a directed graph e.g. follower/followee network. When forming the *Sub Posts Graph*, we form a subgraph of the *All Posts Graph*. This only contains users from the posts in which the candidate keyphrase is mentioned. This way we are able to extract user-level information at the candidate keyphrase level. The way in which we form the *Sub Posts Graph* is the same as [2] which calculates user-interaction features when classifying hashtags as "organic" or "promoted". As discussed, the graph-based features we calculate are recorded in Table S10. A lot of the features are picked on the basis of speed i.e. are computed quickly. Some of the features in the table are quite self-explanatory (e.g. *Num Nodes*, *Num Edges*, *Density* and *Density of Largest Connected Component*), however, some are not. For the "*Degree of each Node*" feature, we find the degree of each node for the *Sub Posts Graph* and then treat the feature as a continuous one - making up 4 univariate time series.[24] For the *Num Nodes in Connected Components* feature, we generate the connected components of the *Sub Posts Graph* and then again treat the feature as a continuous one - making up 4 univariate time series.[25]

Although not discussed in Section 3.4, we also add the *author_premium* field provided by Pushshift to this family of features (i.e. User Based Series). This is one of

[22] Many of the substrings we use in our analysis are of low n-gram range (e.g. 3 grams like "bot") as we end up taking the most common 100 grams

[23] `https://networkx.org/` - last accessed 10/07/2024

[24] `https://networkx.org/documentation/stable/reference/classes/generated/networkx.Graph.degree.html` - last accessed 10/07/2024

[25] `https://networkx.org/documentation/stable/reference/algorithms/generated/networkx.algorithms.components.connected_components.html` - last accessed 10/07/2024

the only personal user-based features provided by Pushshift. We decided to include this feature in this section rather than Appendix A (i.e. Reddit Based Series) as it is an attribute of users.

Table S10. User Features Used in Analysis

**Name**	**Type**	**Num Series**
Username Substrings	str	100
Num Nodes	keyphrase-level	1
Num Edges	keyphrase-level	1
Degree of each Node	cont	4
Num Nodes in Connected Components	cont	4
Density	keyphrase-level	1
Density of Largest Connected Component	keyphrase-level	1
author_premium	bool	2

References

1. Kuehl N. Needmining: Towards analytical support for service design. In: International Conference on Exploring Services Science. Springer; 2016. p. 187–200.

2. Varol O, Ferrara E, Menczer F, Flammini A. Early detection of promoted campaigns on social media. EPJ data science. 2017;6:1–19.

3. Baumgartner J, Zannettou S, Keegan B, Squire M, Blackburn J. The pushshift reddit dataset. In: Proceedings of the international AAAI conference on web and social media. vol. 14; 2020. p. 830–839.

4. To HQ, Nguyen KV, Nguyen NLT, Nguyen AGT. Gender prediction based on vietnamese names with machine learning techniques. In: Proceedings of the 4th International Conference on Natural Language Processing and Information Retrieval; 2020. p. 55–60.

5. Shelton M, Lo K, Nardi B. Online media forums as separate social lives: a qualitative study of disclosure within and beyond Reddit. IConference 2015 Proceedings. 2015;.

6. Kilroy D, Healy G, Caton S. Using Machine Learning to Improve Lead Times in the Identification of Emerging Customer Needs. IEEE Access. 2022;10:37774–37795.

7. Gorwa R, Guilbeault D. Unpacking the social media bot: A typology to guide research and policy. Policy & Internet. 2020;12(2):225–248.

8. Cresci S, Lillo F, Regoli D, Tardelli S, Tesconi M. $ FAKE: Evidence of spam and bot activity in stock microblogs on Twitter. In: Proceedings of the International AAAI Conference on Web and Social Media. vol. 12; 2018.

9. Anderson KE. Ask me anything: what is Reddit? Library Hi Tech News. 2015;32(5):8–11.

10. Li W, Dickinson M. Gender Prediction for Chinese Social Media Data. In: RANLP; 2017. p. 438–445.

11. Kosse R, Schuur Y, Cnossen G. Mixing traditional methods with neural networks for gender prediction. In: Proceedings of the Ninth International Conference of the CLEF Association (CLEF 2018); 2018.

12. Khandelwal A, Swami S, Akhtar SS, Shrivastava M. Gender prediction in english-hindi code-mixed social media content: Corpus and baseline system. Computación y Sistemas. 2018;22(4):1241–1247.
