# Supplementary material for: Prediction of future customer needs using machine learning across multiple product categories
Source: PLoS One. 2024 Aug 26;19(8):e0307180. doi: 10.1371/journal.pone.0307180 (PMC11346667; doi:10.1371/journal.pone.0307180)
Supplement: S11 Appendix — (PDF) [file pone.0307180.s011.pdf]

Appendix K Baseline Parameters

Table S11. Hyper-parameters used in the baseline approach

Parameter Name	Parameter Description	Product Category	Parameter Value	Step Size	Range Values
Gold Standard Subreddit	Subreddit related to the analyzed product	Toothpaste<br>Dog Food<br>Perfume	r/Dentistry<br>r/dogs<br>r/fragrance	x	x
Google Trends Category	Google trend category related to the analyzed product	Toothpaste<br>Dog Food<br>Perfume	Oral & Dental Care<br>Dogs<br>Perfumes & Fragrances	x	x
% Most Similar to Gold Standard Subreddit	Controls the number of posts used in the analysis by excluding posts based on their similarity to the *Gold Standard Subreddit* parameter	All	x	0.01	0.05 - 0.2
Social Media Min Document Frequency	The min doc freq of a keyphrase in a set of posts in order for it to be considered a need	All	x	0.00001	0.00005 - 0.0002
Min Chi Square P-value	The min chi square value a keyphrase must have when it's frequency on Reddit is compared to a reference corpus	All	x	0.01	0.01 - 0.03

The baseline algorithm has many parameters to be able to run it for each category. Table S11 shows these parameters and includes a brief explanation of their role in the algorithm. Although not stated in the baseline, we can broadly split these parameters into two types: a) dynamic and b) static.

The dynamic parameters are searched across multiple different values and are the same for each of the categories analyzed in the baseline. These consist of a) *% Most Similar to Gold Standard Subreddit*; b) *Social Media Min Document Frequency*; and c) *Min Chi Square P-value*. An exhaustive grid search is carried out in the baseline study which tries out multiple values for these 3 parameters to see how it affects the performance of finding future customer needs in the Toothpaste product category [1]. In their evaluation, they didn't want to show that only a specific combination of values for these parameters performs well e.g. when the *% Most Similar to Gold Standard Subreddit* is 0.06, the *Social Media Min Document Frequency* is 0.00009 and the *Min Chi Square P-value* is 0.02. Instead, they report a value range for each of the parameters where the model performs well i.e. not overfitting. All the possible values for a parameter are donated by the Range Values and Step Size columns in Table S11 e.g. the *Min Chi Square P-value* parameter uses the values 0.01, 0.02 and 0.03 as the Range Values is 0.01 - 0.03 and the Step Size is 0.01. As multiple combinations of values are tried out for each parameter we report the mean results for each metric in our

evaluation, as done in the baseline experiment [1]. The mean results for our approach are reported, however for different reasons than the baseline i.e. stochastic processes performed when transforming data not trying out different combinations of parameters (as detailed in Section 4.1). Although these parameter ranges are only found for the Toothpaste category (the only category analyzed in the baseline experiment), we also use these ranges for the other two categories we analyze i.e. Dog Food and Perfume. We do this in the lack of other recommended parameter values for these categories along with the need to do a multi-category baseline comparison.

Different from dynamic parameters, the static parameters are not searched across multiple different values and are different for each of the categories analyzed. These consist of a) *Gold Standard Subreddit* and b) *Google Trends Category*. The *Gold Standard Subreddit* is used in the data reduction step and is defined as "the subreddit which is similar to the product category under analysis" [1]. The baseline uses the subreddit r/Dentistry for the Toothpaste category for this parameter. We follow on from the baseline using the same subreddit for the Toothpaste category. However, we select the subreddits r/fragrance for the Perfume category and r/dogs for the Dog Food category. We do this as these subreddits are highly similar to the product category under analysis (e.g. r/fragrance discusses Perfume products) and due to the lack of the baseline study providing any recommendations for this parameter for these categories. The *Google Trends Category* is used when collecting Google Trends data and impacts the final ranking of keyphrases used in the baseline.[26] The baseline uses the "Oral & Dental Care" category for the Toothpaste product category. We follow along with the baseline for this value. However, for the Perfume and Dog Food product categories, we use the "Perfumes & Fragrances" and "Dogs" respectively. We do this as they are similar to the corresponding product categories.

References

1. Kilroy D, Healy G, Caton S. Using Machine Learning to Improve Lead Times in the Identification of Emerging Customer Needs. IEEE Access. 2022;10:37774–37795.

[26] `https://github.com/pat310/google-trends-api/wiki/Google-Trends-Categories` - last accessed 10/07/2024
