# Supplementary material for: Prediction of future customer needs using machine learning across multiple product categories
Source: PLoS One. 2024 Aug 26;19(8):e0307180. doi: 10.1371/journal.pone.0307180 (PMC11346667; doi:10.1371/journal.pone.0307180)
Supplement: S12 Appendix — (PDF) [file pone.0307180.s012.pdf]

Appendix L Comprehensive Evaluation Metrics

The following shows the precision and recall results for the F1 scores for Tables 5, 9 and 13. This is performed to provide a more comprehensive view of the evaluation metrics instead of just the F1 score. Other metrics such as accuracy are not recorded as the number of keyphrase instances to be included in the classification task is influenced by the nature of the approach i.e. the number of keyphrases to include depends on the text processing and keyphrase selection part of the pipeline (Section 3.2). Therefore, including it would not provide useful insights and it could lead to misinterpretations of the metric.

The tables show that the machine learning approach obtains much higher precision than recall scores when optimised for the F1 metric (as detailed in Section 4.1). This is seen across almost all the tables in this appendix. For this particular application, precision is a good metric to score highly on as it would allow potential users of this model to trust its output more than if it were low.

Performing this comprehensive analysis has given more useful insights into how the model is working. This could help with model debugging for future work.

Table S12. Binary Classification Evaluation showing the mean Precision scores (rounded to 3 decimal places) for the One Category and baseline approaches. This table shows the precision score from Table 5.

**Category**	**Method**					
	One Category	*Baseline*				
Toothpaste	0.314	*5*	*10*	*15*	*20*	*50*
		0.201	0.139	0.134	0.115	0.76
		100	*250*	*500*	*750*	*1000*
		0.064	0.057	0.049	0.044	0.041
		1250	*1500*	*1750*	*2000*	*2500*
		0.039	0.035	0.032	0.031	0.029
Dog Food	0.326	*5*	*10*	*15*	*20*	*50*
		0	0.004	0.024	0.034	0.047
		100	*250*	*500*	*750*	*1000*
		0.041	0.05	0.045	0.043	0.041
		1250	*1500*	*1750*	*2000*	*2500*
		0.04	0.038	0.037	0.034	0.032
Perfume	0.432	*5*	*10*	*15*	*20*	*50*
		0	0.007	0.009	0.018	0.057
		100	*250*	*500*	*750*	*1000*
		0.055	0.046	0.043	0.046	0.047
		1250	*1500*	*1750*	*2000*	*2500*
		0.046	0.043	0.04	0.037	0.031

Table S13. Binary Classification Evaluation showing the mean Recall scores (rounded to 3 decimal places) for the One Category and baseline approaches. This table shows the recall score from Table 5.

**Category**	**Method**					
	One Category	*Baseline*				
Toothpaste	0.063	*5*	*10*	*15*	*20*	*50*
		0.006	0.009	0.012	0.014	0.024
		100	*250*	*500*	*750*	*1000*
		0.039	0.087	0.149	0.201	0.255
		1250	*1500*	*1750*	*2000*	*2500*
		0.297	0.325	0.349	0.378	0.449
Dog Food	0.076	*5*	*10*	*15*	*20*	*50*
		0	0	0.002	0.004	0.012
		100	*250*	*500*	*750*	*1000*
		0.022	0.066	0.118	0.169	0.217
		1250	*1500*	*1750*	*2000*	*2500*
		0.265	0.303	0.336	0.35	0.353
Perfume	0.083	*5*	*10*	*15*	*20*	*50*
		0	0.001	0.001	0.003	0.025
		100	*250*	*500*	*750*	*1000*
		0.049	0.101	0.193	0.306	0.421
		1250	*1500*	*1750*	*2000*	*2500*
		0.513	0.574	0.624	0.655	0.667

Table S14. Binary Classification Evaluation showing the mean Precision and Recall Scores (rounded to 3 decimal places) for the One Category and Multiple Category approaches across the Seen Testing Categories. This table shows the precision and recall scores from Table 9.

**Category**	**Method**			
	One Category		*Multiple Category*	
	**Precision**	**Recall**	**Precision**	**Recall**
Dog Food	0.326	0.076	0.204	0.104
Eyeliner	0.197	0.056	0.264	0.053
Lip Balm	0.513	0.056	0.322	0.103
Nail Polish	0.388	0.061	0.310	0.084
Perfume	0.432	0.083	0.383	0.054
Shampoo	0.275	0.082	0.299	0.087
Toothpaste	0.314	0.063	0.262	0.086

Table S15. Binary Classification Evaluation showing the mean Precision and Recall Scores (rounded to 3 decimal places) for the Multiple Category approach for the Unseen Testing Categories. This table shows the precision and recall scores from Table 13.

**Category**	**Method**	
	Multiple Category	
	**Precision**	**Recall**
Beer	0.261	0.063
Cereal	0.331	0.071
Coffee	0.291	0.063
Cookie	0.364	0.051
Pizza	0.269	0.091
Popcorn	0.279	0.079
Soda	0.339	0.042
Soup	0.440	0.049
