# Supplementary material for: Prediction of future customer needs using machine learning across multiple product categories
Source: PLoS One. 2024 Aug 26;19(8):e0307180. doi: 10.1371/journal.pone.0307180 (PMC11346667; doi:10.1371/journal.pone.0307180)
Supplement: S13 Appendix — (PDF) [file pone.0307180.s013.pdf]

Appendix M Seen & Unseen Categories F1 Score Distribution

To further visualize the fact that the F1 scores for the Seen and Unseen Testing Categories for the Multiple Category approach don't differ much from each other, a kernel density estimate plot of these scores are plotted in Fig S3 using the python library seaborn.[27] This plot is used as it's more visually intuitive than a histogram [1, 2]. For the *bandwidth* (a key parameter used to smooth the plot produced by kernel density estimation [2]), we use the default value in seaborn i.e. *bandwidth*=1. We do this across all the 10 runs for each category, therefore recording 80 F1 scores for the Unseen Testing Categories (i.e. 8 categories multiplied by 10 runs) and 70 F1 scores for the Seen Testing Categories (i.e. 7 categories multiplied by 10 runs). The plot shows that although the results from the Seen Testing Categories are higher than the Unseen Testing Categories, they don't deviate much from each other. Hence, we can conclude that the Multi-Task Learning (MTL) approach can still predict future customer needs on a category it has not seen during training with relatively similar performance to ones it has seen during training.

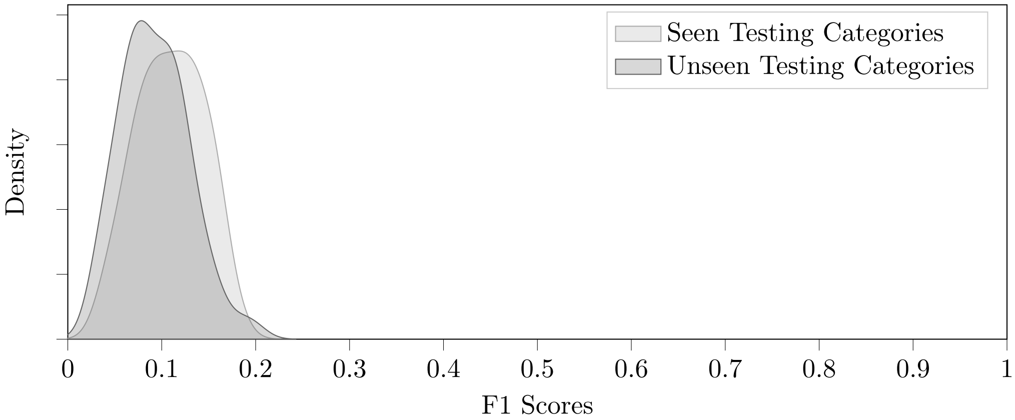

Fig S3. Seen vs Unseen Testing Category F1 result distribution - the seen and unseen categories predict with relatively similar accuracy. The x-axis shows the F1 scores while the y-axis shows the density of the distribution.

References

1. Węglarczyk S. Kernel density estimation and its application. In: ITM Web of Conferences. vol. 23. EDP Sciences; 2018. p. 00037.

2. Chen YC. A tutorial on kernel density estimation and recent advances. Biostatistics & Epidemiology. 2017;1(1):161–187.

[27] `https://seaborn.pydata.org/generated/seaborn.kdeplot.html` - last accessed 10/07/2024
