# Supplementary material for: Prediction of future customer needs using machine learning across multiple product categories
Source: PLoS One. 2024 Aug 26;19(8):e0307180. doi: 10.1371/journal.pone.0307180 (PMC11346667; doi:10.1371/journal.pone.0307180)
Supplement: S14 Appendix — (PDF) [file pone.0307180.s014.pdf]

Appendix N Seen & Unseen Categories Mean Precision and Recall Score Distribution

To further visualize the fact that the List Mean Precision and List Recall scores for the Seen and Unseen Testing Categories for the Multiple Category approach don't differ much from each other, multiple kernel density estimate plots of these scores across each value of K (i.e. number of submitted keyphrases) are shown in Fig S4.[28] For each plot, we do this across all 10 runs for each category. The plots show the results don't deviate much from each other. Hence, we can conclude that the Multi-Task Learning (MTL) approach can still predict future customer needs on a category it has not seen during training with relatively similar performance to ones it has seen during training.

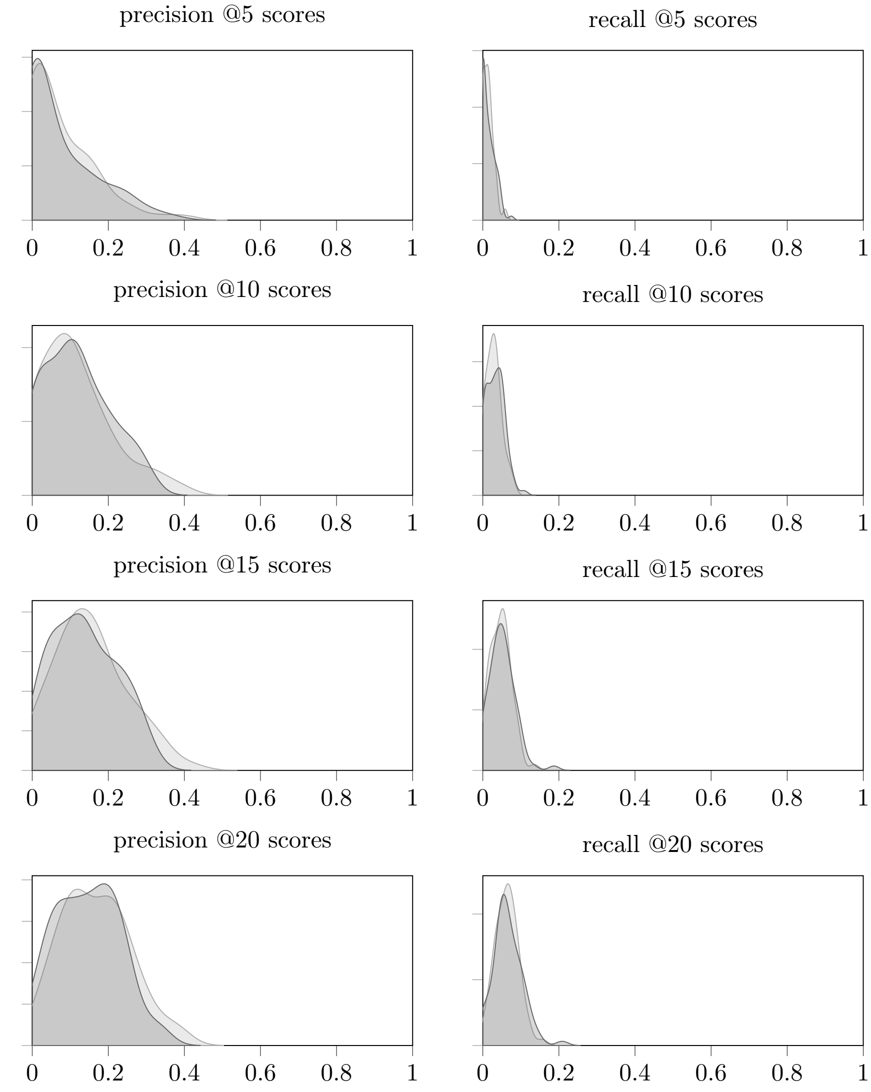

Fig S4. Multiple Category approach for the Seen/Unseen results of List Evaluation. For each plot, the x-axis shows the results while the y-axis shows the density of the values.

[28]This is shown instead of a histogram for the same reasons as detailed in Appendix M (more visually intuitive). The same library as in Appendix M is also used to generate the plots i.e seaborn.
