# Supplementary material for: Prediction of future customer needs using machine learning across multiple product categories
Source: PLoS One. 2024 Aug 26;19(8):e0307180. doi: 10.1371/journal.pone.0307180 (PMC11346667; doi:10.1371/journal.pone.0307180)
Supplement: S15 Appendix — (PDF) [file pone.0307180.s015.pdf]

Appendix O Lead Times of Future Customer Needs

In this section, we observe the lead times the social media model detects future customer needs in the Trending Customer Needs (TCN) dataset. To do this, we obtain all the keyphrases the Multi-Task Learning (MTL) model correctly predicts as future customer needs i.e. in the TCN dataset 1-3 years in the future. These keyphrases are then checked to see how far out they are from the date of prediction to the date they are in the TCN dataset. This is done across all the 15 product categories tested in the analysis and across all 10 runs of each category - each category tested in the analysis is run 10 times (as described in Section 4.1). In our analysis, we only record the date in the TCN dataset nearest to when the keyphrase from the MTL model was predicted e.g. if the model predicted "charcoal" on 2018-01-01 and "charcoal" appeared in the TCN dataset on 2019-01-01, 2020-06-01 and 2021-03-01 we only consider the gap to be 1 year as 2018-01-01 is 1 year away from 2019-01-01. When checking how far out the keyphrases are from the month of prediction to the date they are in the TCN dataset, we also include dates that are closer than 1 year away even if they are detected as future customer needs by the model e.g. if the model predicted "coconut" on 2018-01-01 and "coconut" appeared in the TCN dataset on 2018-06-01, 2019-01-01, 2020-06-01 and 2021-03-01 we only consider the gap to be 5 months as 2018-01-01 is 6 months away from 2018-06-01 and is the first date nearest from the prediction.

Fig 8 (in the main text) shows a kernel density estimation plot of these lead times.[29] Although a lot of these future customer needs are detected before 5 months there are a lot found past 2 years in advance. Such lead times would be highly beneficial for companies to identify before these needs start to become popular in the marketplace.

[29] This is shown instead of a histogram for the same reasons as detailed in Appendix M and N (more visually intuitive). The same library as in Appendix M and N is also used to generate the plots i.e seaborn.
