# Supplementary material for: Prediction of future customer needs using machine learning across multiple product categories
Source: PLoS One. 2024 Aug 26;19(8):e0307180. doi: 10.1371/journal.pone.0307180 (PMC11346667; doi:10.1371/journal.pone.0307180)
Supplement: S16 Appendix — (PDF) [file pone.0307180.s016.pdf]

Appendix P Future Optimizations

In this section, we discuss the limitations of the validation procedure which hinders the approach at finding future customer needs with increased performance.

As discussed earlier in the evaluation (i.e. Section 4.1), we validate the output probability threshold parameter of the model by finding its value which optimizes the F1 score at predicting future customer needs. The data used to train and validate the model comes from 2014-01-01 to 2014-12-31 while the data used to test the model comes from 2018-01-01 to 2018-12-31. These splits in the training and testing times are required so that no instance overlap occurs (discussed in Section 4.1). When applying the validated probability threshold parameter value (estimated from 2014) to the test data (in 2018), we can investigate how well it performs for each category by comparing the F1 score it generates to the F1 score represented by the optimal probability threshold parameter value which can be found in the test data. This optimal probability threshold parameter value can be found the same way the validated threshold parameter value is found, however, it requires the ground truth label which is not provided in real-world scenarios, hence why we don't already use it during testing. To show how well the assumptions hold, we record the difference between the optimal F1 score found in the test data compared to the F1 score estimated during validation for each category.

These differences are shown in Table S16 which tracks the mean difference (rounded to 3 decimal places) for each category each month between 2018-01-01 to 2018-12-01 across the 10 runs of the algorithm. There is quite a lot of room for improvement in the validation parameter estimation approach used in this study with some mean differences being as high as 5% F1. An area where this could be improved is in the way in which we split the data into training and validation sets, which is not conventional given the nuances of the training data having overlapping time series (described in Section 4.1). Improving this process is an area of future work. As discussed earlier in the evaluation (Section 4.1), the probability threshold is the only hyper-parameter tuned in this study for computational/time purposes. Tuning other hyper-parameters (e.g. inputs into MINImally RandOm Convolutional KErnel Transform (MINIROCKET)) is also an area of future work that would allow for increases in model performance.

Table S16. Mean difference (rounded to 3 decimal places) between the optimal F1 score on the test set and the chosen F1 score in the validation set for the Probability Threshold parameter

**Category**	**Mean F1 Difference**
Beer	0.032
Cereal	0.029
Coffee	0.034
Cookie	0.042
Dog Food	0.024
Eyeliner	0.035
Lip Balm	0.036
Nail Polish	0.042
Perfume	0.046
Pizza	0.027
Popcorn	0.036
Shampoo	0.034
Soda	0.054
Soup	0.050
Toothpaste	0.021
