# Supplementary material for: Prediction of future customer needs using machine learning across multiple product categories
Source: PLoS One. 2024 Aug 26;19(8):e0307180. doi: 10.1371/journal.pone.0307180 (PMC11346667; doi:10.1371/journal.pone.0307180)
Supplement: S17 Appendix — (PDF) [file pone.0307180.s017.pdf]

Appendix Q Performance Graphs

In this section, we plot the results of the Binary Classification Evaluation using some alternative visualizations/metrics other than the F1 score (used in Section 4.3 and Section 4.4). Specifically, we plot the Precision-Recall (PR) Curve and the Receiver Operating Characteristic (ROC) Curve - both typically used to show the performance of binary classification at a range of probability thresholds [1]. The PR Curve is built by plotting precision-recall pairs obtained using different thresholds from a probabilistic (or other continuous-output) classifier [1]. The ROC Curve is built the same way using thresholds from a classifier, however by plotting true-false positive rate pairs instead. When plotting, we also report the Area Under Curve (AUC) for both the PR Curve (i.e. AUC PR [2]) and ROC Curve (i.e. AUC ROC [2]). We do this as these metrics have been used a lot to compare models [2] while providing a different view of our results other than the F1, which has been used extensively in our study. Although we plot both the PR Curve and the ROC Curve, the PR Curve is known to be better for binary classification scenarios where the data is imbalanced [3] (as in our study). The main reason for this is due to the inclusion of True Negatives in the False Positive Rate for the ROC Curve and the mindful avoidance of this in the PR Curve. By including the True Negatives in the calculation, importance is given to situations where the classifier predicts an instance to be the negative class when it is in fact the negative class, which is highly common in imbalanced classification tasks where the focus should instead be on predicting the positive class. This leads to a random baseline classifier achieving a AUC ROC score of 0.5 regardless of the distribution of the output class, whereas the AUC PR for the random classifier moves with the distribution of the output class [3].

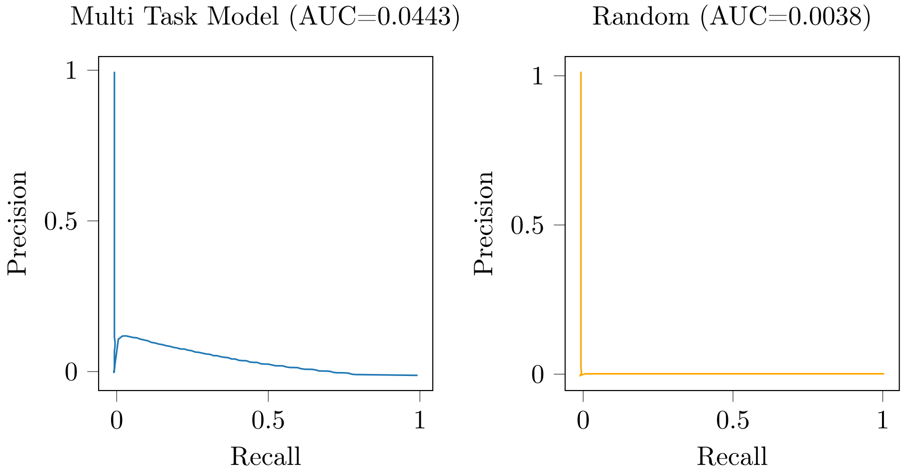

Fig S5. Precision Recall Curve for the Multi-Task Learning (MTL) Model and a Random Classifier. The PR AUC score (rounded to 4 decimal places) is also shown for each classifier.

Fig S5 shows the PR Curve for the MTL model and a random classifier. The AUC for each of the PR curves is also shown in the figure (rounded to 4 decimal places). Here the random classifier generates probability values from a continuous uniform distribution in the range of 0 to 1.[30] Specifically, it generates these values from the output of the keyphrase selection process (i.e. Section 3.2) and therefore assumes no Multivariate Time Series Classification (MTSC) takes place. This random classifier generates an AUC PR score of 0.0038 which approximates the ratio of the initial class

[30] `https://docs.scipy.org/doc/numpy-1.15.0/reference/generated/numpy.random.random.html` - last accessed 10/07/2024

distribution i.e. 260:1 (detailed in Section 4.1). As discussed in the AUC PR literature, this approximation is expected [3]. The MTL model has a AUC PR score of 0.0443 which outperforms the random classifier by some margin (≈11.5 times better). This shows that a significant amount of learning is taking place during MTSC (i.e. Section 3.3, Section 3.4 and Section 3.5), as the random classifier aimlessly picks keyphrases the MTL approach learns features on Reddit from. Of additional note in the figure is that the precision and recall both drop to zero across some probability thresholds in the PR Curve for the MTL model. This is because there are a number of keyphrases which are in the Trending Customer Needs (TCN) dataset (i.e. ground truth), however, are never selected as candidate keyphrases during the Text Processing & Keyphrase Selection process (i.e. Section 3.2). These are accounted for when performing our evaluation, however, are given a predicted probability score of 0 as they never have a chance of being predicted as a positive label. Although barely visible in the figure, the same occurs for the random classifier as these keyphrases are not chosen during keyphrase selection.

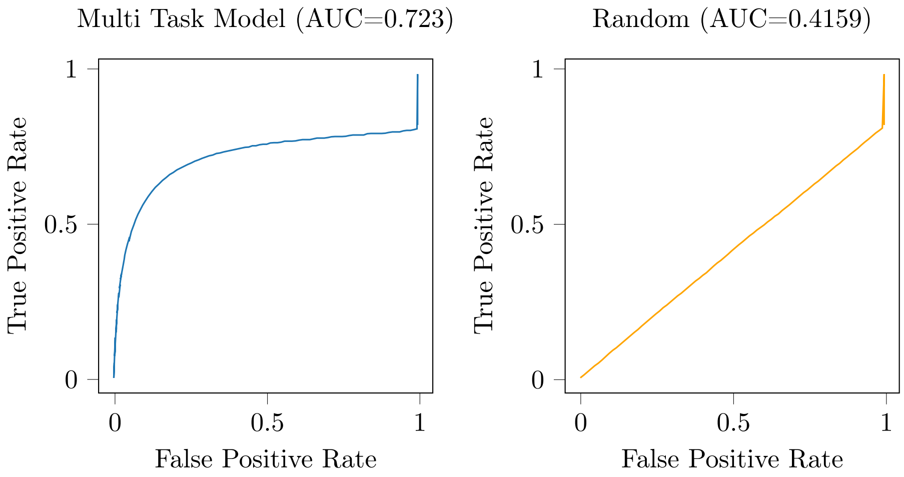

Fig S6. Receiver Operating Characteristic Curve for the MTL Model and a Random Classifier. The ROC AUC score (rounded to 4 decimal places) is also shown for each classifier.

Fig S6 shows the ROC Curve for the MTL model and a random classifier. The AUC for each of the ROC curves is also shown in the figure (rounded to 4 decimal places). The same random classifier used for the PR curve is used in this figure. The random classifier achieves a AUC ROC score of 0.4159, which is contradictory to the literature that random achieves a score of 0.5 [3]. This occurs as there are a certain number of ground truth keyphrases that are never found in the keyphrase selection process (as discussed earlier in this section). This is the reason why the ROC curves for both the MTL and random classifiers dramatically shoot up at the threshold 0. As with the PR Curve, the ROC curve for the MTL model (0.723) is better than the random classifier (0.4159). However, as discussed, the figure does not paint the full picture of the real difference between the two classifiers.

References

1. Boyd K, Eng KH, Page CD. Area under the precision-recall curve: point estimates and confidence intervals. In: Machine Learning and Knowledge Discovery in Databases: European Conference, ECML PKDD 2013, Prague, Czech Republic, September 23-27, 2013, Proceedings, Part III 13. Springer; 2013. p. 451–466.

2. Davis J, Goadrich M. The relationship between Precision-Recall and ROC curves. In: Proceedings of the 23rd international conference on Machine learning; 2006. p. 233–240.

3. Saito T, Rehmsmeier M. The precision-recall plot is more informative than the ROC plot when evaluating binary classifiers on imbalanced datasets. PloS one. 2015;10(3):e0118432.
