# Supplementary material for: Prediction of future customer needs using machine learning across multiple product categories
Source: PLoS One. 2024 Aug 26;19(8):e0307180. doi: 10.1371/journal.pone.0307180 (PMC11346667; doi:10.1371/journal.pone.0307180)
Supplement: S18 Appendix — (PDF) [file pone.0307180.s018.pdf]

Appendix R Misclassifications

Table S17. The top 10 predicted keyphrases the Multi-Task Learning (MTL) model thinks are future customer needs (ranked by prediction probability output), however aren't (i.e. are not in the Trending Customer Needs (TCN) dataset 1-3 years in the future)

Category	Keyphrases
Beer	garlic, onion, corn, grape, tomato, fried, ginger, baked, liquid, funky
Cereal	flax, olive, mashed, fatty, whey, dressing, nutritional, walnuts, melon, cheesecake
Coffee	unsweetened, avocado, dairy, tomato, sour, blueberry, extract, veggie, olive, pepper
Cookie	roasted, toasted, grape, marshmallow, beef, tart, garlic, crust, glaze, buttery
Eyeliner	precision, voluminous, amazonian, sweet, cosmetic, pomade, enhance, facial, glossy, clay
Lip Balm	moisturising, smooth, hydrate, healthy, neutral, antioxidant, soft, facial, agave, delicious
Nail Polish	acrylic, cream, enamel, finish, lemon, solvent, berry, neutral, acetate, wine
Perfume	aromatic, amber, apricot, bergamot, oily, hydrating, sandalwood, grapefruit, intense, frankincense
Pizza	almond, coconut, pudding, roasted, shredded, vanilla, pretzel, biscuit, fatty, balsamic
Popcorn	roasted, savory, hummus, cucumber, baked, nuts, tortilla, broccoli, watermelon, nutritional
Soda	almond, creamy, potassium, optional, spicy, cocoa, coconut, unsweetened, maple, stevia
Soup	fatty, nutritional, cashew, radish, eggplant, rosemary, toasted, steamed, rotisserie, crispy

This section aims to highlight instances where the model made the largest prediction errors. We do this to illustrate where there are some inherent problems associated with it or where it can be improved so to better understand future work directions to improve it. Specifically, we try to understand where the model is wrong in misclassifying a keyphrase as a "future customer need" when it isn't one and vice versa i.e. not a "future customer need" when it is one. In order to find these instances we make use of the probability output omitted by the MTL model for each keyphrase and its associated ground truth label in the TCN dataset. Table S17 records the most probable keyphrases the MTL model predicts as future customer needs (i.e. highest prediction probability output) which are not future needs (i.e. did not appear in the TCN dataset 1-3 years in the future). This therefore shows the keyphrase instances the MTL model most thinks are future customer needs, however aren't. Table S18 then records the most probable keyphrases the MTL predicts are not future customer needs (i.e. lowest prediction probability output) which in fact are future needs (i.e. appear in the TCN dataset 1-3 years in the future). This shows the keyphrase instances the MTL model most thinks are not future customer needs, however are. For this table, in order to actually show the most probable keyphrases the MTL model predicts are not future needs we show the keyphrases which have the lowest probability output omitted by the model not just ones given a probability score of zero for the purposes of our evaluation (detailed in

Appendix Q). For the information in both Table S17 and S18, we report the keyphrases with the highest mean predicted probability output across all runs of the model i.e. we run the MTL approach 10 times for all product categories (as described in Section 4.1).

In Table S17 (i.e. keyphrases wrongly predicted as future customer needs), there appears to be only one dominant issue where the model is making a mistake i.e. misclassifying. This is that the model predicts irrelevant keyphrases for a product category e.g. "garlic" for the Beer category, "beef" for the Cookie category or "voluminous" for the Eyeliner category. This occurs due to the way in which the MTL model is trained on a wide variety of product categories. Through training this way it learns the characteristics of a future customer need rather than needs for its own category. Therefore, if a keyphrase meets the criteria of being a future customer need according to the model, it will be predicted in the positive class e.g. mentioned often enough on social media (document frequency), has a phrase embedding which is shared in the same embedding space as previous positive instances (phrase embedding). This is thus the reason why there appears to be seemingly strange keyphrases for a particular category predicted with high confidence by the model.

Table S18. The top 10 predicted keyphrases the model thinks are not future customer needs (ranked by prediction probability output), however are (i.e. are in the TCN dataset 1-3 years in the future)

Category	Keyphrases
Beer	india pale, recycled, barley malt, top ferment, alcohol free, pilsen, kosher, light, alcohol, preservative
Cereal	freeze dry, artificial color, red blood, palm oil, sustainable, recycle, straight, refined sugar, saturated fat, fatigue
Coffee	freeze dry, rounded, halal, low acidity, compostable, single origin, protect, aluminium, full bodied, silky
Cookie	trans fat, wafers, sustainable, palm oil, trans, halal, colouring, sticks, corn syrup, egg
Eyeliner	vitamin e, animal testing, precise application, jojoba oil, sensitive eye, resin, felt tip, water resistant, mineral oil, drying
Lip Balm	dead skin cell, moisture loss, animal testing, gently exfoliate, sweet almond oil, regenerate, revive, stimulate, sweet almond, penetrate
Nail Polish	high shine, full coverage, animal testing, flat brush, easy application, dibutyl phthalate, highly pigmented, high gloss, phthalate, nail enamel
Perfume	perfumed body, halal, animal testing, softness, recycled, vegetarian, cedar wood, raw material, sustainable, femininity
Pizza	stuffed crust, recyclable, hard, wood fire, palm, gluten free, dry tomato, sustainable, fry, pepperoni
Popcorn	palm oil, handmade, halal, air pop, sunflower oil, trans fat, hot air, add sugar, gmo, cholesterol
Soda	halal, fat, calorie, cream, sugar, cola, red, zero, alcohol, fruit
Soup	pea, add sugar, chicken bone broth, palm, bpa, halal, extra virgin olive oil, stew, chicken, egg

In Table S18 (i.e. keyphrases not predicted as future customer needs), there similarly appears to only be one issue occurring. This is that certain keyphrases don't have the predicted probability output of being a future customer need that might be expected. This can said for almost every keyphrase in the table e.g. "alcohol free" for Beer, "high shine" for Nail Polish, "cream" for Soda or "pea" for Soup. This is almost the opposite problem of the first issue mentioned in Table S17 which is that certain irrelevant

keyphrases are predicted for a product category. However, part of this problem arises for the same first issue in Table S17, which is the fact that the model is finding it difficult to learn keyphrases associated with its own product category due to the way in which the model is trained during the MTL process.

There are most likely a lot of ways that the discussed issue of predicting keyphrases which are not relevant to a category can be remedied. One way could be to add more Machine Learning (ML) features which relate a keyphrase to its associated category. Some of these existing types of features include comparing a keyphrase's frequency to a background corpus (detailed in Appendix B). However, some additional ways of guiding the model to get to know that a keyphrase is related to a domain would be useful in mitigating these errors [1–3]. This could make the approach resistant to these types of issues even when employing the MTL technique of learning customer need keyphrases from other categories i.e. as each keyphrase would have a relatedness score to its respective category.

References

1. Alami Merrouni Z, Frikh B, Ouhbi B. Automatic keyphrase extraction: a survey and trends. Journal of Intelligent Information Systems. 2020;54:391–424.

2. Hasan KS, Ng V. Automatic keyphrase extraction: A survey of the state of the art. In: Proceedings of the 52nd Annual Meeting of the Association for Computational Linguistics (Volume 1: Long Papers); 2014. p. 1262–1273.

3. Merrouni ZA, Frikh B, Ouhbi B. Automatic keyphrase extraction: An overview of the state of the art. In: 2016 4th IEEE international colloquium on information science and technology (CiSt). IEEE; 2016. p. 306–313.
