# Supplementary material for: Prediction of future customer needs using machine learning across multiple product categories
Source: PLoS One. 2024 Aug 26;19(8):e0307180. doi: 10.1371/journal.pone.0307180 (PMC11346667; doi:10.1371/journal.pone.0307180)
Supplement: Attachment — Submitted filename: Response to Reviewers.pdf [file pone.0307180.s020.pdf]

We thank the reviewers for their feedback and suggestions. We have attempted to address all comments (discussed below); the following details the changes made in correspondence with the reviewers and editors comments. We note that these changes are also highlighted in the submitted manuscript for convenience.

## Reviewer 1

**1. Author mentioned in Abstract section it is a First study while same approach already available previously.**

The abstract previously said that it was the first study to use the TCN dataset, not the first study in the literature. We have changed abstract text to make it clearer that this is the first study to use the TCN dataset.

**2. Author mainly focused Dataset, features, and metrics in whole paper, so that it is missing Methods clarification properly, kindly re organized paper.**

This is handled in Section 4, and we have improved the introduction of Section 4 to make this clearer.

**3. The author must focus on the small typos, uses of punctuation and English level throughout the manuscript.**

The manuscript has been thoroughly proofread, changes arising from this have been made throughout the manuscript and are highlighted in pink.

**4. Author should mention Which type of Machine Learning Algorithm used with proper explanation.**

A discussion of the multivariate time series algorithm MINIRocket is provided. We have altered the text to clarify that this is indeed a supervised ML-based algorithm. This is provided in Section 4.5.

**5. I will suggest author to use more exiting method for comparison of the proposed method to show the result.**

This is the only other approach previously used to predict future customer needs at a category level (e.g. mobile phones), many other works have performed similar tasks at the product level (e.g. iPhone). This manner of evaluation has been accepted in 2022, and since then we have not identified other works trying to solve the problem at the product category level to compare against. If the reviewer is aware of alternatives, we would happily include them in the manuscript.

The evaluation methodology itself is informed by recent literature of how keyphrases are assessed in ranked lists (taken largely from the event detection on social media literature [1])

and binary classifiers (taken from the recent/impactful literature in imbalanced binary classification [2]).

**6. In Conclusion part Author explained very lengthy it should be concluded in one or two paragraph, as this paper required (It should be minimized)**

The conclusion of the article is 2 paragraphs. The future work/scope of the article is a further 2 paragraphs (see reviewer comment 8).

**7. Need to modify references also, most of the references are too old.**

5 new references from 2023 and 2 new references from 2022 have been added. The following illustrates a distribution of the references for the current manuscript which shows a large number of references are from the last 7 years.

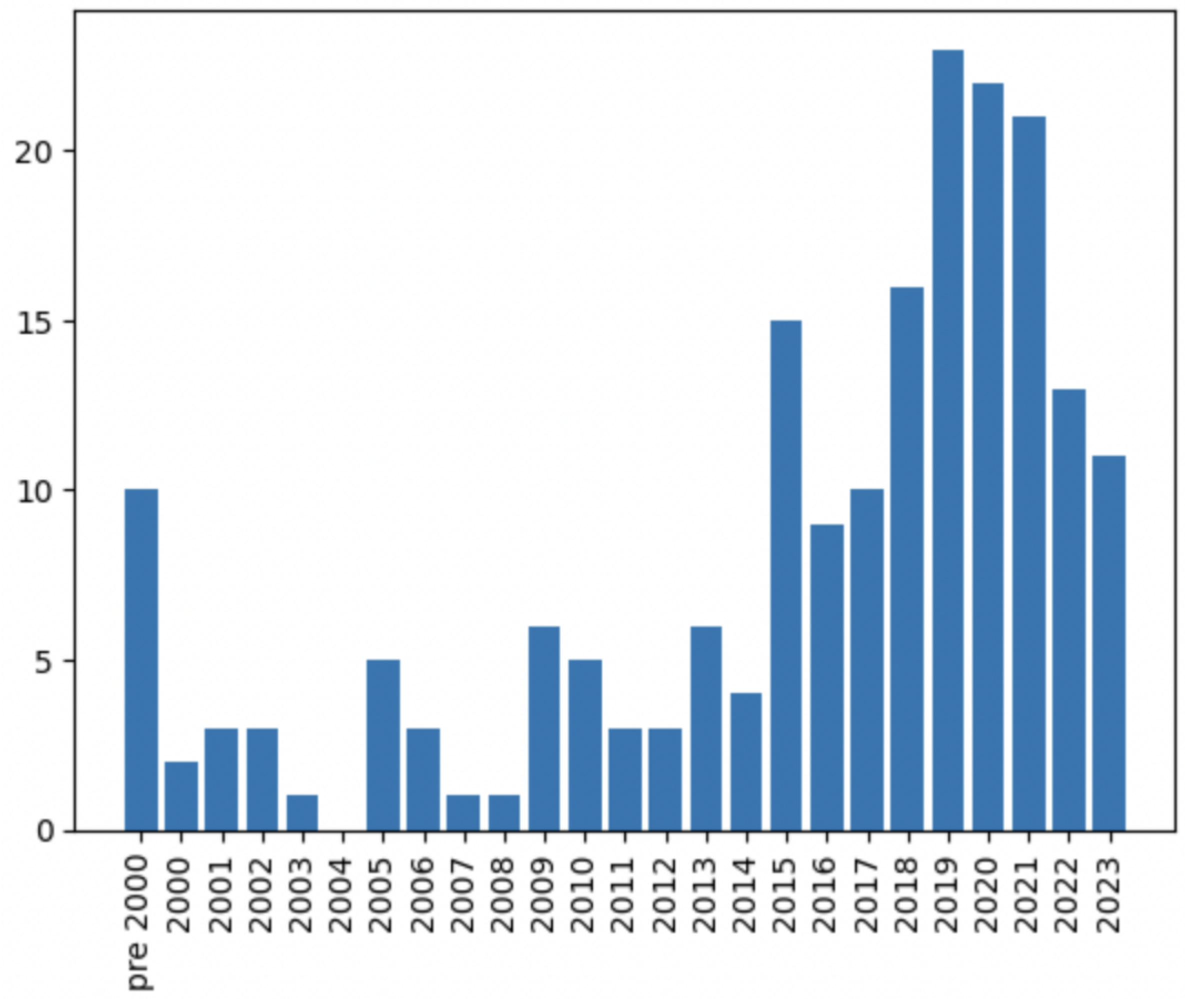


**8. Author Should add Future scope of this Article.**

The future scope/work of this article is included in the conclusion. We have renamed the “Conclusion” section to “Conclusion & Future Work” for more clarity.

## Reviewer 2

**1. The authors wrote "The challenging task of predicting future customer needs is performed better than previous studies" but at the same time they are claiming that no previous studies worked on predicting future customer needs. Kindly clarify it.**

In the evaluation, the study is compared to a previously published baseline approach which performs the same task in this paper using a different method. The authors could not find any specific claims stating that no previous studies predict future customer needs in the manuscript. Due to the potential confusion various text changes were made throughout the manuscript to clarify that there are other studies predicting future customer needs.

**2. The entire manuscript needs to be scanned to address the varied language issues. For example, on line 257 "is how other approaches differ on"**

The manuscript has been thoroughly proofread, text changes arising from this have been made throughout the manuscript and are highlighted in pink.

**3. There should be a precise name of Algorithm 1. Further, all the steps of this algorithm should be clearly elaborated.**

We respond to this point together with point 4 below.

**4. The sub-algorithms of Algorithm 1 are absent in the study. For example, getDataForCategory() These algorithms should also be written with clear and well defined instructions so that the potential reader may understand them.**

We understand the reviewer's concerns for giving a precise name to "Algorithm 1". However in accordance with how previous PLOS papers have named algorithms using numbers [3-7], we have kept it the same e.g. Algorithm 1, Algorithm 2 etc. We have however provided a description for the algorithm beside its name (highlighted) to address the reviewer's comment. We have also expanded the text to provide additional commentary concerning how the sub-algorithms work within the text.

**5. For the evaluation, the SOTA metrics are missing. They must be used.**

Metrics from the previous baseline study performing the same task have been used in this study when evaluating lists of keyphrases i.e. List Mean Precision and List Recall. Additionally, the most popular ML metric for imbalanced binary classification was used when evaluating on the instance level i.e. F1 measure. These metrics are considered SOTA for the task addressed in this paper. Section 5.2 provides a discussion concerning why these metrics are chosen.

**6. Lastly, article is too much lengthy. It must be made succinct.**

We have attempted to locate material we view as supplementary and moved this to the appendix. When revising the manuscript we have endeavoured to shorten the manuscript further, for example, we have removed section 2. However, we feel that shortening it any further may lead to problems where readers cannot understand the study given the depth of interdisciplinary technical content throughout the manuscript.

# Academic Editor

**1. When submitting your revision, we need you to address these additional requirements. Please ensure that your manuscript meets PLOS ONE's style requirements, including those for file naming. The PLOS ONE style templates can be found at https://journals.plos.org/plosone/s/file?id=wjVg/PLOSOne_formatting_sample_main_body.pdf and https://journals.plos.org/plosone/s/file?id=ba62/PLOSOne_formatting_sample_title_authors_affiliations.pdf**

We have checked the additional requirements and made relevant changes.

**2. In your Methods section, please include additional information about your dataset and ensure that you have included a statement specifying whether the collection and analysis method complied with the terms and conditions for the source of the data.**

We note that the data we used emanated from a previous publication, and we cite this paper in the manuscript as well as providing a repository link. We also confirm that we have permission to use the data and that the collection and analysis methodology complies with the terms and conditions of the data owner. We have added this as a footnote to the manuscript (footnote number 11).

**3. Please note that PLOS ONE has specific guidelines on code sharing for submissions in which author-generated code underpins the findings in the manuscript. In these cases, all author-generated code must be made available without restrictions upon publication of the work. Please review our guidelines at https://journals.plos.org/plosone/s/materials-and-software-sharing#loc-sharing-code and ensure that your code is shared in a way that follows best practice and facilitates reproducibility and reuse.**

We provide a link to the companion GitHub repository for this manuscript at the end of the introduction.

**4. Thank you for stating the following in the Acknowledgments Section of your manuscript: This publication has emanated from research supported in part by a grant from Science 1599 Foundation Ireland under Grant number 18/CRT/6183. For the purpose of Open 1600 Access, the author has applied a CC BY public copyright licence to any Author 1601 Accepted Manuscript version arising from this submission.**

**We note that you have provided funding information that is not currently declared in your Funding Statement. However, funding information should not appear in the**

**Acknowledgments section or other areas of your manuscript. We will only publish funding information present in the Funding Statement section of the online submission form.**
**Please remove any funding-related text from the manuscript and let us know how you would like to update your Funding Statement. Currently, your Funding Statement reads as follows:**
**DK, 18/CRT/6183, Science Foundation Ireland, https://www.sfi.ie/, No**

**Please include your amended statements within your cover letter; we will change the online submission form on your behalf.**

We have removed the acknowledgements from the manuscript. We were asked by our funding agency to include this statement in the acknowledgement section of manuscripts, however, if this doesn't meet PLOS guidelines it can be left out. We have no additional information to provide for the online submission form.

**5. Thank you for stating the following in your Competing Interests section:**
**"No"**

**Please complete your Competing Interests on the online submission form to state any Competing Interests. If you have no competing interests, please state "The authors have declared that no competing interests exist.", as detailed online in our guide for authors at http://journals.plos.org/plosone/s/submit-now**
**This information should be included in your cover letter; we will change the online submission form on your behalf.**

We have no competing interests.

**6. Please note that in order to use the direct billing option the corresponding author must be affiliated with the chosen institute. Please either amend your manuscript to change the affiliation or corresponding author, or email us at plosone@plos.org with a request to remove this option.**

The corresponding author is affiliated with the chosen institute. Science Foundation Ireland (SFI) funds University College Dublin (UCD). The corresponding author has a UCD email address not a SFI email address, which is where this misunderstanding may have arisen.

**7. Please include your full ethics statement in the 'Methods' section of your manuscript file. In your statement, please include the full name of the IRB or ethics committee who approved or waived your study, as well as whether or not you obtained informed written or verbal consent. If consent was waived for your study, please include this information in your statement as well.**

We have added a footnote (footnote number 10) in the methodology section specifying the following: "This work received an ethics waiver from the UCD Human Research Ethics Committee – Sciences (HREC-LS) under ref LS-E-20-81-Kilroy-Caton."

**8.Please include captions for your Supporting Information files at the end of your manuscript, and update any in-text citations to match accordingly. Please see our**

**Supporting Information guidelines for more information: http://journals.plos.org/plosone/s/supporting-information.**

We have checked this and confirm it to be the case.


[1] Aiello LM, Petkos G, Martin C, Corney D, Papadopoulos S, Skraba R, Göker A, Kompatsiaris I, Jaimes A. Sensing trending topics in Twitter. IEEE Transactions on multimedia. 2013 Jun 6;15(6):1268-82.
[2] Brownlee J. Imbalanced classification with Python: better metrics, balance skewed classes, cost-sensitive learning. Machine Learning Mastery; 2020.
[3] Boughorbel S, Jarray F, El-Anbari M. Optimal classifier for imbalanced data using Matthews Correlation Coefficient metric. PloS one. 2017 Jun 2;12(6):e0177678.
[4] Shen W, Le S, Li Y, Hu F. SeqKit: a cross-platform and ultrafast toolkit for FASTA/Q file manipulation. PloS one. 2016 Oct 5;11(10):e0163962.
[5] Zheng D, Yang G, Li X, Wang Z, Liu F, He L. An efficient algorithm for computing attractors of synchronous and asynchronous Boolean networks. PloS one. 2013 Apr 9;8(4):e60593.
[6] Zhang L, Liu L, Yang XS, Dai Y. A novel hybrid firefly algorithm for global optimization. PloS one. 2016 Sep 29;11(9):e0163230.
[7] Abraham G, Inouye M. Fast principal component analysis of large-scale genome-wide data. PloS one. 2014 Apr 9;9(4):e93766.
