# Supplementary material for: Prediction of future customer needs using machine learning across multiple product categories
Source: PLoS One. 2024 Aug 26;19(8):e0307180. doi: 10.1371/journal.pone.0307180 (PMC11346667; doi:10.1371/journal.pone.0307180)
Supplement: Attachment — Submitted filename: Response to Reviewers.pdf [file pone.0307180.s021.pdf]

We thank the reviewers for their feedback and suggestions. We have attempted to address all comments (discussed below); the following details the changes made in correspondence with the reviewers and editors comments. We note that these changes are also highlighted in the submitted manuscript for convenience.

Reviewer 1

No further comments.

Reviewer 2

1. The entire manuscript needs to be scanned to fix the typos and other grammar issues.

The manuscript has been thoroughly proofread again, changes arising from this have been made throughout (highlighted in pink).

2. Algorithm 1 needs to be renamed. Further, normally, Algorithm and its caption are written on the top of the algorithm instructions.

We have renamed Algorithm 1 to “Social Media Algorithm”. The caption for it is also now written on top of the algorithm instructions.

3. Results of the present study must be compared with the state of the art.

We have added a new section (Section 4.5) to address this. Here we compare our algorithm to available SOTA methods.

4. The results must be tested with the comprehensive validation metrics.

This is a valid point. We have added a new Appendix to address this suggestion (Appendix L). This appendix shows the precision and recall results from the sole F1 metric which is recorded in the main paper (for the binary based evaluation). This provides a more comprehensive view of performance and allows the paper to illustrate different aspects of performance that a user may value. We thank the reviewer for this suggestion and feel that it has increased both the transparency of analysis and yielded a better more rounded discussion of model performance.
