# Supplementary material for: Prediction of future customer needs using machine learning across multiple product categories
Source: PLoS One. 2024 Aug 26;19(8):e0307180. doi: 10.1371/journal.pone.0307180 (PMC11346667; doi:10.1371/journal.pone.0307180)
Supplement: Attachment — Submitted filename: reivew.docx [file pone.0307180.s019.docx]

1. The authors wrote “The challenging task of predicting future customer needs is performed better than previous studies” but at the same time they are claiming that no previous studies worked on predicting future customer needs. Kindly clarify it.
2. The entire manuscript needs to be scanned to address the varied language issues. For example, on line 257 “is how other approaches differ on”
3. There should be a precise name of Algorithm 1. Further, all the steps of this algorithm should be clearly elaborated.
4. The sub-algorithms of Algorithm 1 are absent in the study. For example, **getDataForCategory()**

These algorithms should also be written with clear and well defined instructions so that the potential reader may understand them.

5. For the evaluation, the SOTA metrics are missing. They must be used.
6. Lastly, article is too much lengthy. It must be made succinct.
